# Transcriptomic Analysis Reveals the Protective Effects of Empagliflozin on Lipid Metabolism in Nonalcoholic Fatty Liver Disease

**DOI:** 10.3389/fphar.2021.793586

**Published:** 2021-12-21

**Authors:** Yuting Ma, Chengxia Kan, Hongyan Qiu, Yongping Liu, Ningning Hou, Fang Han, Junfeng Shi, Xiaodong Sun

**Affiliations:** ^1^ Department of Endocrinology and Metabolism, Affiliated Hospital of Weifang Medical University, Weifang, China; ^2^ Branch of Shandong Provincial Clinical Research Center for Diabetes and Metabolic Diseases, Weifang, China; ^3^ Clinical Research Center, Affiliated Hospital of Weifang Medical University, Weifang, China; ^4^ Department of Pathology, Affiliated Hospital of Weifang Medical University, Weifang, China

**Keywords:** empaglifiozin, NAFLD, obesity, lipid, transcriptome

## Abstract

Empagliflozin is a novel type of sodium-glucose cotransporter two inhibitor with diverse beneficial effects in the treatment of nonalcoholic fatty liver disease (NAFLD). Although empagliflozin impacts NAFLD by regulating lipid metabolism, the underlying mechanism has not been fully elucidated. In this study, we investigated transcriptional regulation pathways affected by empagliflozin in a mouse model of NAFLD. In this study, NAFLD was established in male C57BL/6J mice by administration of a high-fat diet; it was then treated with empagliflozin and whole transcriptome analysis was conducted. Gene expression levels detected by transcriptome analysis were then verified by quantitative real-time polymerase chain reaction, protein levels detected by Western Blot. Differential expression genes screened from RNA-Seq data were enriched in lipid metabolism and synthesis. The Gene Set Enrichment Analysis (GSEA) results showed decreased lipid synthesis and improved lipid metabolism. Empagliflozin improved NAFLD through enhanced triglyceride transfer, triglyceride lipolysis and microsomal mitochondrial β-oxidation. This study provides new insights concerning the mechanisms by which sodium-glucose cotransporter two inhibitors impact NAFLD, particularly in terms of liver lipid metabolism. The lipid metabolism-related genes identified in this experiment provide robust evidence for further analyses of the mechanism by which empagliflozin impacts NAFLD.

## Introduction

Nonalcoholic fatty liver disease (NAFLD) is a common chronic liver disease, characterized by a broad spectrum of clinical manifestations (e.g., hepatic steatosis, fibrosis, cirrhosis, and hepatocellular carcinoma) (Rinella, 2015; Manne et al., 2018). Importantly, NAFLD affects one-quarter of adults worldwide (Zhou et al., 2019). Metabolic diseases (e.g., obesity and type 2 diabetes) have been associated with greater NAFLD risk in previous studies (Perumpail et al., 2017). Considering the increasing morbidity of obesity and diabetes, the prevalences of NAFLD and advanced liver disease are expected to increase (Cotter and Rinella, 2020). Lipid metabolism disorders have essential roles in NAFLD occurrence and progression (Mato et al., 2019; Di et al., 2021). For example, hepatic steatosis (a typical symptom of NAFLD) results from lipid acquisition that exceeds lipid disposal (Ipsen et al., 2018). Considering that the liver has a central role in lipid synthesis and metabolism, it is important to study drug prevention and treatment of NAFLD from the perspective of liver lipid metabolism. Recently, SGLT2i has become a focus because of beneficial effect on reduced serum transaminase activities (Seko et al., 2018). Furthermore, several pilot studies showed SGLT2i had a significant reduction in liver transaminases, body weight, and the fatty liver index in NAFLD patients (Komiya et al., 2016; Seko et al., 2017). Therefore, the efficacy of SGLT2 inhibitor on NASH/NAFLD can be expected.

Sodium-glucose cotransporter 2 (SGLT2) inhibitors are effective hypoglycemic drugs that function by inhibiting glucose reabsorption in proximal renal tubules. SGLT2 inhibitors improve glycemic status in patients with type 2 diabetes; they also contribute to weight loss and body fat reduction (Szekeres et al., 2021). Multiple studies have reported that SGLT2 inhibitor treatments reduce serum cholesterol and triglyceride (TG) levels (Hayashi et al., 2017; Calapkulu et al., 2019); they also increase lipolysis, liver fatty acid oxidation, and ketogenesis (Osataphan et al., 2019). Empagliflozin (EMP), a novel anti-hyperglycemic agent (Supplementary Figure S1), is a type of SGLT2 inhibitor (Frampton, 2018). Although EMP was designed as a hypoglycemic drug, it also has an important effect on lipid metabolism and has been shown to reduce liver fat in NAFLD (Kuchay et al., 2018). Studies by Xu et al. revealed that EMP contributes to weight loss through increased fat utilization and browning. Furthermore, reductions of obesity-related inflammation by EMP have been associated with M2 macrophage polarization (Xu et al., 2017; Xu et al., 2019). Additionally, recent studies indicated SGLT2i decreased body weight and lipid profiles, including total cholesterol (TC) and TG (Al-Sharea et al., 2018; Matsubayashi et al., 2020; Liu et al., 2021). However, there is a few researches illustrating how EMP regulated the progress of TG accumulation and associated triglyceride molecules.

Triglyceride molecule is the main form of storage and transport of fatty acids in cells and plasma. Fatty acid activation is catalyzed by long-chain acyl-CoA synthetase (ACSL) family of enzymes and microsomal G3P acyltransferase (GPAT) enzymes catalyzed G3P pathway, which is the main way to synthesize TG (Cohen and Fisher, 2013). The triglyceride transfer protein (MTP) combined with TG to form VLDL particles (Cohen and Fisher, 2013). Furthermore, adipose triglyceride lipase (ATGL, also known as PNPLA2), a rate-limiting step of TG lipolysis in adipocytes was activated by the comparative gene identification-58 (CGI-58) (Lass et al., 2006). Liver specific resection of CGI-58 can lead to NAFLD phenotype in mice, including hepatic steatosis and hepatic fibrosis (Guo et al., 2013). The patatin-like phospholipase domain-containing 3 (PNPLA3) has a genetic association with NALFD, and mice showed accumulation of inactive PNPLA3 in lipid droplets and increased steatosis in high-fat diet feeding (Smagris et al., 2015). Hepatic TG can be oxidized by multiple pathways. Mitochondrial β-oxidation is the main route in hepatocytes (Musso et al., 2009). The expression of genes including mitochondrial β-oxidation is regulated largely by PPARα activity. PGC1α and BAF60a form a complex to regulate activity of PPARα transcriptional in the liver (Li et al., 2008).

Moreover, with the rapid development of RNAseq technology, transcriptome analysis of NAFLD is increasing. Previous investigations suggested lipid metabolism plays a major role in NAFLD (Xu et al., 2021). However, the molecular mechanisms underlying EMP-mediated NAFLD protection of hepatic lipid metabolism have not been fully elucidated. Here, we investigated signaling pathways impacted by EMP in a mouse model of NAFLD and explored the mechanisms and triglyceride molecules underlying the effects of EMP on liver lipid metabolism and synthesis.

## Materials and Methods

### Animal Model and Drug Administration

All animal experiments in this study were approved by the Animal Ethics Committee of Weifang Medical University and adhered to national guidelines for experiments involving laboratory animals. Six-week-old male C57BL/6J mice (weight, 21 ± 0.8 g) were purchased from Pengyue Co., Ltd. (Jinan, China). All mice were housed under a 12-h day/night cycle at constant temperature (approximately 22°C) and suitable humidity (45–70%). As previous study (Han et al., 2020), the CT group (n = 8) was fed a regular diet (320 kcal per 100 g) (Pengyue Co., Ltd. Jinan, China), and the HFD (n = 8) and EMP groups (n = 8) were fed a high-fat diet (54.05% fat, 15.10% protein, and 30.85% carbohydrate; 529.8 kcal per 100 g) (Fanbo Biotechnology Co., Ltd., Shanghai, China) for 20 weeks. After the 12 weeks of feeding, the EMP group underwent further intragastric administration of EMP (10 mg/kg/day) for 8 weeks, whereas CT and HFD groups received same volume of saline for 8 weeks. After successive drug administration for 8 weeks, the mice were given 2% barbiturate sodium (40 mg/kg, ip) after 12 h of fasting. Body composition of the mice were measured by Body Composition Analysis (Bruker Minispec LF50, German). Blood samples were collected. Livers were also rapidly isolated and cut into several small pieces; two pieces of liver were fixed with formalin, then subjected to hematoxylin-eosin (HE) staining and Oil Red O staining. Plasma triglycerides and liver triglycerides were measured using triglyceride assay kit from Jiancheng (Nanjing, China). The remaining liver tissues were stored at −80°C for RNA extraction.

### Total RNA Extraction, cDNA Library Construction, and RNA Sequencing

Total RNA was extracted and purified from liver tissue using TRIzol, in accordance with the manufacturer’s instructions (Invitrogen, United States). Bioanalyzer 2,100 (Agilent, United States) and NanoDrop ND-1000 (NanoDrop, United States) equipment were used to assess total RNA quantity and quality. Poly (A) RNA was purified twice using Dynabeads Oligo (dT) (Thermo Fisher, United States). The resulting RNA was fragmented using a Magnesium RNA Fragmentation Module (NEB, United States) at 94°C for 5–7 min. RNA was used as a template for cDNA synthesis by SuperScript™ II Reverse transcriptase (Invitrogen). Single- or dual-index adapters were ligated to the fragments; AMPureXP beads were used for size selection. The mean insert size in the final cDNA library was 300 ± 50 bp. RNA-Seq was performed by Illumina NovaSeq™ 6,000 (LC Bio Technology Co., Ltd. China); 150-bp paired-end sequences were generated.

### Quantification of RNA and Analysis of Differential Expression

FastQC was used to assess sequence quality. After removal of low-quality reads, the remaining 150-bp paired-end sequences were reassembled and mapped to the *Mus musculus* genome by HISAT2 (Kim et al., 2015). The transcripts were merged from sequences by StringTie (Pertea et al., 2015). To assure quality of RNA, we finally chosen five samples per group for further study. Genes with low expression levels were discarded when their minimum expression thresholds were below one count per million (CPM) in all 15 samples. The expression data were normalized by trimmed mean of M-values (TMM) using the edgR package in Bioconductor (Robinson et al., 2010); counts of sequences were transformed to log_2_(CPM) values for subsequent estimation of gene expression levels. Differentially expressed genes (DEGs) were identified using the limma package in R (Ritchie et al., 2015) with the following settings: fold change >2 or < −2, and *p* < 0.01.

### Analysis of Enrichment, PPI and GSEA

Gene Ontology (GO) (Young et al., 2010) and Kyoto Encyclopedia of Genes and Genomes (KEGG) (Kanehisa et al., 2008) analyses of DEGs were conducted using the clusterProfiler package in R (Yu et al., 2012). DEGs were integrated with mouse protein-protein interaction (PPI) networks using STRING. PPI networks were analyzed by Cytoscape 3.8.0 (Shannon et al., 2003). Gene Set Enrichment Analysis (GSEA) was used for additional pathway analyses; forward regression was then performed (Subramanian et al., 2005).

### Real-Time Quantitative PCR

DEGs related to lipid metabolism and synthesis were subjected to validation by qPCR. Total RNA was extracted as described above for RNA-Seq analysis. cDNA was obtained using the PrimerScript™ RT reagent Kit with gDNA Eraser (TaKaRa, Japan). qPCR was performed using TB Green^®^ Premix Ex Taq™ (TaKaRa). Primers of target genes were synthesized by Sangon Biotech (China); Table 1 shows the sequences of primers used in this study. All relative expression levels of RNA were calculated based on Ct values, then normalized to the levels of β-actin (Liu et al., 2011) as follows: 2 [ΔCt = Ct (target genes) − Ct (β-actin)].

**TABLE 1:** The sequence of the primers for qRT-PCR.

Gene	Forward primer (5′-3′)	Reverse primer (5′-3′)
ACSL3	AAC​CAC​GTA​TCT​TCA​ACA​CCA​TC	AGT​CCG​GTT​TGG​AAC​TGA​CAG
ACSL5	TCC​TGA​CGT​TTG​GAA​CGG​C	CTC​CCT​CAA​TCC​CCA​CAG​AC
CGI-58	TGG​GGT​TTT​CCT​GAG​CGA​C	GGT​TAA​AGG​GAG​TCA​ATG​CTG​C
Cyp2g1	GCA​CTT​TGT​TTG​TCT​TGC​CTG	TCC​CAA​AAA​CGG​TAT​TGG​TGT​G
Fam131c	CCT​CTC​TGG​ATG​ACG​AAG​AAC​T	TGT​CCT​GAA​GGT​AGA​TGC​TCT​C
Fdps	GGA​GGT​CCT​AGA​GTA​CAA​TGC​C	AAG​CCT​GGA​GCA​GTT​CTA​CAC
GPAT1	ACA​GTT​GGC​ACA​ATA​GAC​GTT​T	CCT​TCC​ATT​TCA​GTG​TTG​CAG​A
Hmgcs1	AAC​TGG​TGC​AGA​AAT​CTC​TAG​C	GGT​TGA​ATA​GCT​CAG​AAC​TAG​CC
Hr	CCC​CTG​TGA​ACG​GCA​TTG​T	CCC​CTC​CAA​AAG​GGA​GCA​G
HSL	CCA​GCC​TGA​GGG​CTT​ACT​G	CTC​CAT​TGA​CTG​TGA​CAT​CTC​G
MTP	CTC​TTG​GCA​GTG​CTT​TTT​CTC​T	GAG​CTT​GTA​TAG​CCG​CTC​ATT
PGC-1α	TAT​GGA​GTG​ACA​TAG​AGT​GTG​CT	CCA​CTT​CAA​TCC​ACC​CAG​AAA​G
PNPLA3	TCA​CCT​TCG​TGT​GCA​GTC​TC	CCT​GGA​GCC​CGT​CTC​TGA​T
PPARα	AGA​GCC​CCA​TCT​GTC​CTC​TC	ACT​GGT​AGT​CTG​CAA​AAC​CAA
Rdh1	GTC​ATG​GGC​CGA​ATG​TCT​TTC	GCC​TGT​CAC​TAC​TTG​TCA​CAC​A
Smpd3	TTT​GCC​TTT​CTC​GGG​TTC​ATC	TTG​TCT​TCT​AGC​CGG​GAG​TAG
Sox9	GAG​CCG​GAT​CTG​AAG​AGG​GA	GCT​TGA​CGT​GTG​GCT​TGT​TC
Sqle	ATA​AGA​AAT​GCG​GGG​ATG​TCA​C	ATA​TCC​GAG​AAG​GCA​GCG​AAC
β-actin	GGC​TGT​ATT​CCC​CTC​CAT​CG	CCA​GTT​GGT​AAC​AAT​GCC​ATG​T

### Western Blot

Total protein from liver tissues were lysed in ice-cold Lysis buffer (Whole Cell Lysis Assay, KeyGEN, China), and the total protein concentration was quantified by a BCA Protein Assay Kit (Solarbio, China). Equal amounts of protein were electrophoresed on 10% SDS-PAGE gels and transferred to PVDF membranes. After blocking with 5% BSA, the membranes were incubated with primary antibodies (FDPS, HMGCS1 1:1,000, Proteintech) overnight at 4°C. After washing, membranes were soaked in the secondary antibodies (Anti-rabbit IgG HRP-linked, 1:5,000, Anti-mouse IgG HRP-linked, 1:5,000) for 1 h β-actin was used as an internal control. Immunoblots were then visualized by enhanced chemiluminescence (ECL) using ImageQuant LAS 5000 (GE Healthcare) and analyzed using ImageJ.

### Oil Red O Staining and H&E Staining

Mice were anesthetized by intraperitoneal injection of 2% sodium pentobarbital and 4% buffered neutral formalin solution was used to fix their liver tissues for 24 h. Tissue sections were subjected to H&E staining and Oil Red O staining, in accordance with standard protocols. The images were obtained using a microscope (CKX53, Olympus).

### Statistics

Statistical analysis of this study was conducted by GraphPad Prism 7.0. Normality test was performed by Shapiro-Wilk normality test. Normally distributed data were presented as the means ± SEM. One-way ANOVA followed by Dunnett’s *post-hoc* test was performed when data involved in all three groups. Abnormally distributed data are displayed as the median and interquartile intervals, and the distributions between groups were compared with the non-parametric Kruskal-Wallis test. *p* values <0.05 were considered significant.

## Results

### Effects of EMP on Mouse Body Weight and Liver Pathology

**FIGURE 1:**
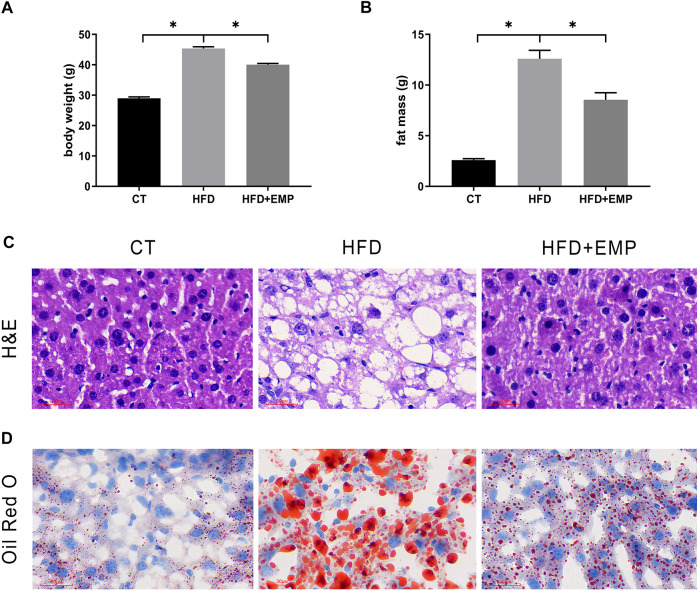
Effect of EMP on mice. The impact of EMP on body weight **(A)** and fat mass **(B)**. Liver histological examination with H&E (200×) **(C)** and Oil Red O staining **(D)**. Data are presented as means ± SEM, *p < 0.05, Scale bar: 30 μm.

All mice were acclimated for 1 week, then randomly separated into CT, HFD, and EMP groups. However, the CT + EMP group is not included in our study. Because our previous study had reported EMP does not have an effect on body weight, fat mass, plasma lipids (Sun et al., 2020). Also, a previous study consistent with our study indicated EMP does not alter liver metabolism and biochemical parameters (Petito-da-Silva et al., 2019). Therefore, we did not establish the CT + EMP group. After 20 weeks of high-fat diet treatment, the weight and fat mass were significantly higher in the HFD and EMP groups than in the CT group. After 8 weeks of intragastric administration of EMP, the weight and fat mass were decreased in the EMP group, compared with the HFD group **(** Figure 1A, B, Table 2 **)**. To access the liver metabolism caused by HFD, we measured alanine aminotransferase (ALT), aspartate aminotransferase (AST) and plasma TG levels. The results showed EMP alleviated increased liver injury with reducing AST, ALT and plasma TG levels (Table 2). Liver changes were observed by H&E and Oil Red O staining **(** Figures 1C,D **)**. Liver tissue structure was normal in the CT group, with distinguishable edges and clear outlines. HFD mice showed prominent diffuse hepatic steatosis with nuclear condensation, cytoplasmic looseness, and increased lipid contents; all of these abnormalities were ameliorated by EMP treatment (Figure 1).

**TABLE 2:** Biometric and blood parameters of rats in the studied groups.

Group	CT	HFD	EMP
Initial body weight (g)	22.17 ± 0.23	22.03 ± 0.19	21.73 ± 0.30
Final body weight (g)	28.97 ± 0.45	45.37 ± 0.58^a^	40.00 ± 0.42^b^
Initial fat mass (g)	1.28 ± 0.03	1.29 ± 0.01	1.26 ± 0.03
Final fat mass (g)	2.59 ± 0.15	12.59 ± 0.84^a^	8.55 ± 0.69^b^
FBG (mmol/L)	5.23 ± 0.93	7.21 ± 1.39	6.85 ± 0.63
triglycerides (mg/dl)	34.25 ± 6.23	52.40 ± 4.78^a^	50.06 ± 6.06
ALT (U/L)	40.10 ± 1.97	159.7 ± 18.86^a^	45.3 ± 3.52^b^
AST (U/L)	45.23 ± 6.41	78.06 ± 9.33^a^	31.2 ± 3.79^b^

Data are shown as mean ± SEM.

a *p* < 0.05 vs CT.

b *p* < 0.05 vs HFD.

### Identification of Differentially Expressed Genes Across Disease and Drug

**FIGURE 2:**
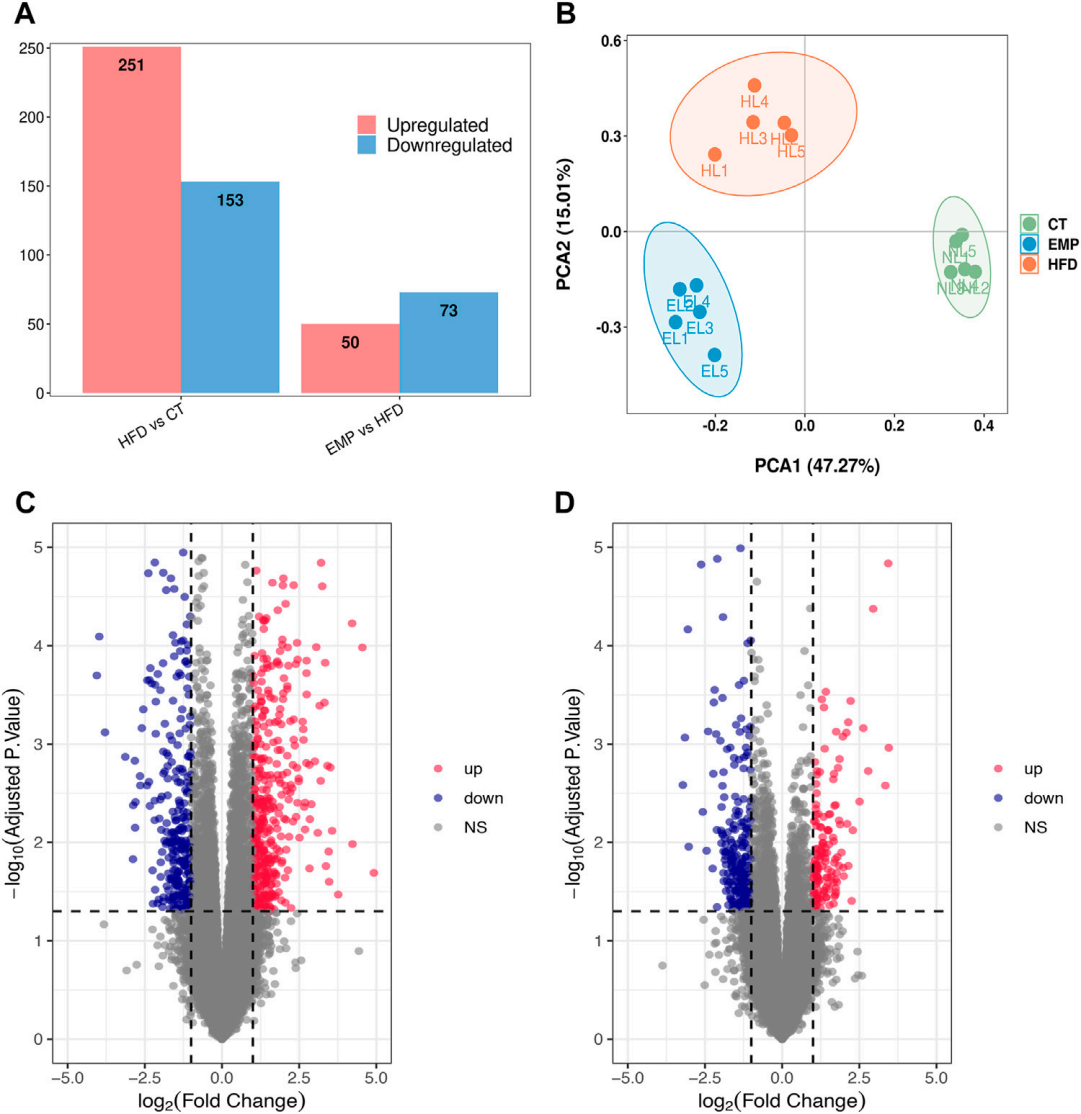
Comparison of transcriptome profiling. **(A)** The gene number of up-regulation and down-regulation in HFD/CT and EMP/HFD. **(B)** PCA analysis of three groups. **(C)** The volcano plots for the distribution of differently expressed genes between HFD and CT group. Blue represents a down-regulation in expression, red represents upregulation and gray represents no significance when compared with control. **(D)** The volcano plots for the distribution of differently expressed genes between EMP and HFD group.

**FIGURE 3:**
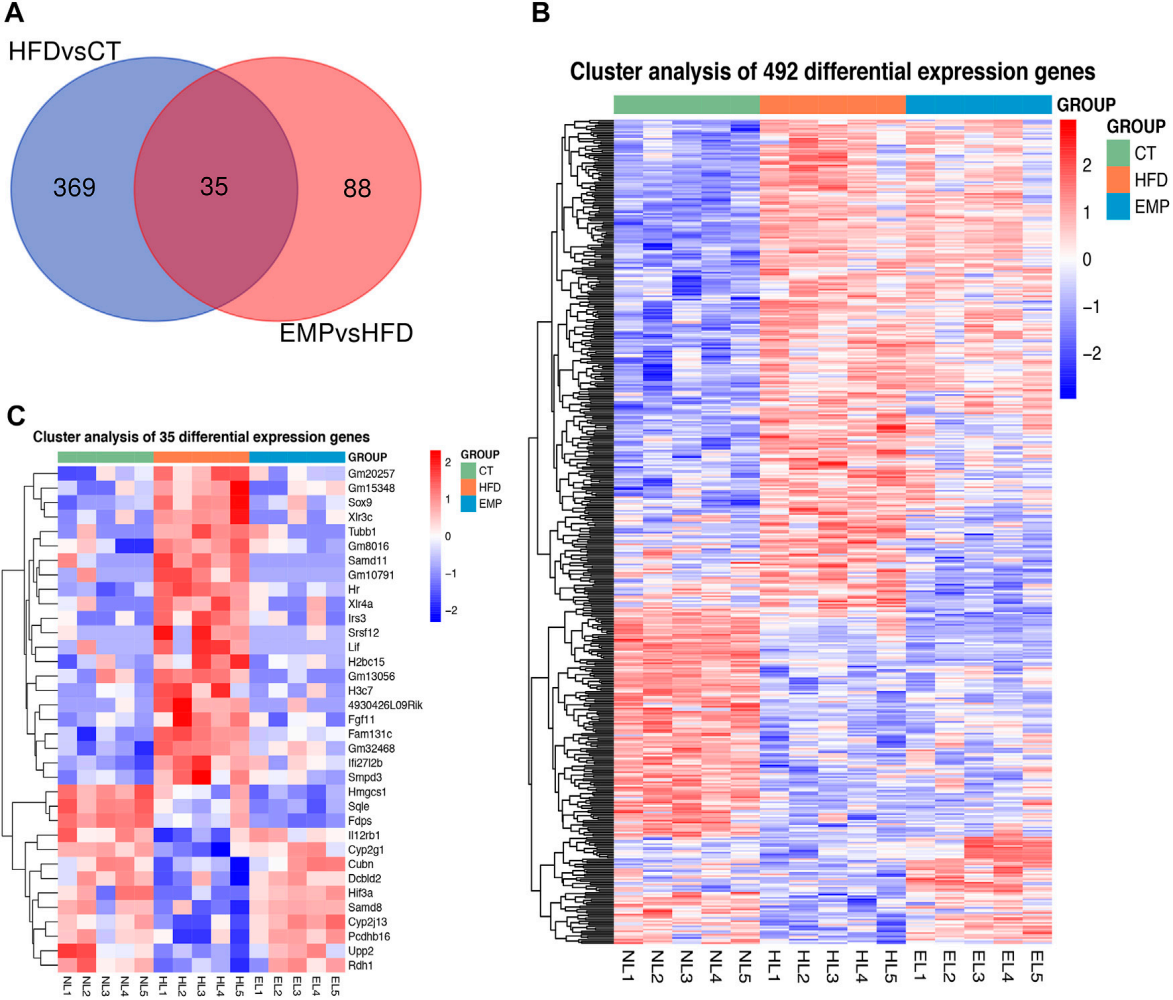
The heatmap representing alteration of hepatic union **(A)** and shared **(B)** genes expression of HFD/CT and EMP/HFD. **(C)** The Venn diagram shows an overlap among the HFD/CT and EMP/HFD genes.

Here, we performed RNA-Seq using liver tissue from NAFLD model mice that had received EMP to explore the mechanisms underlying the effects of EMP on lipid metabolism and synthesis. We first investigated the liver tissue transcriptome profile in mice with regular and high-fat diets to determine gene expression changes during NAFLD. In total, 404 DEGs were observed in the HFD group, compared with the CT group; these consisted of 251 upregulated genes and 153 downregulated genes (Figures 2A,C). To understand how EMP treatment affects NAFLD, we compared transcriptome profiles between the HFD and EMP groups. Compared with the HFD group, 123 DEGs were observed in the EMP group; these consisted of 50 upregulated genes and 73 downregulated genes (Figures 2A,D). We investigated repeatability among samples using principal components analysis (PCA). The PCA results were displayed in a two-dimensional image format (Figure 2B), showing the degrees of separation among samples and groups. Notably, spatial separation trends were observed between HFD and CT groups, as well as between EMP and HFD groups. The findings indicated significant differences in gene expression patterns among the three groups; sample correlation was high within each group. The union and shared DEGs (492 and 35 genes) were displayed in the Venn diagram (Figure 3A). The gene expression profiles of union and shared DEGs exhibited apparent gene expression variations among the CT, HFD, and EMP groups (Figures 3B,C).

### GO and KEGG Pathway Analysis

**FIGURE 4:**
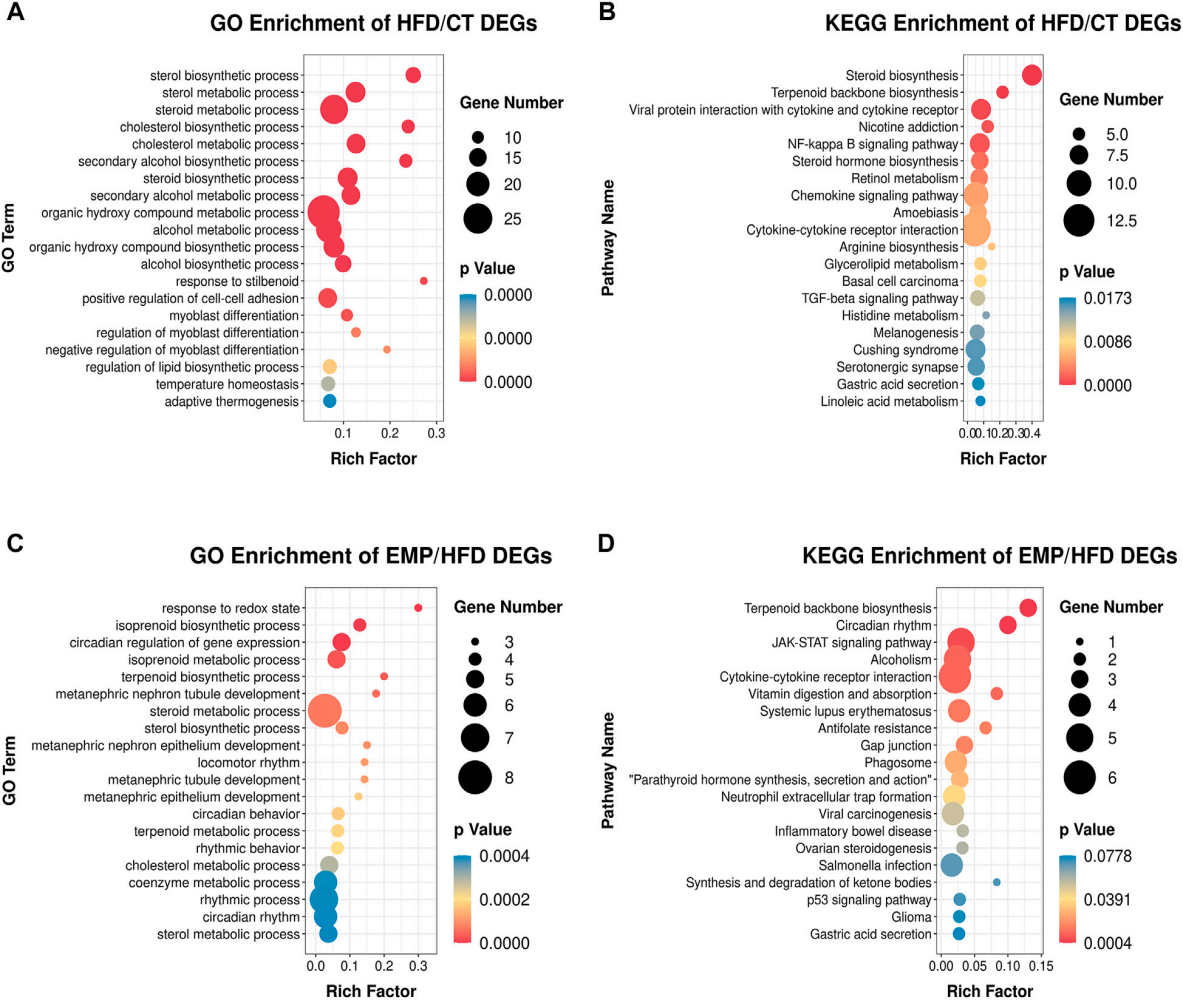
Main GO terms and pathways enriched by DEGs between HFD and CT group, **(A)** GO terms; **(B)** KEGG pathways. Enrichment of GO and pathways based on DEGs between EMP and HFD group, **(C)** GO terms; **(D)** KEGG pathways. Gene Number represents the number of the differential expressed genes enriched in each term.

The expression levels of 404 genes were significantly changed in the HFD group compared with the CT group. GO enrichment analysis showed that the DEGs affected by HFD were mainly involved in the “sterol biosynthetic process,” “sterol metabolic process,” “steroid metabolic process,” “cholesterol biosynthetic process,” “cholesterol metabolic process,” “secondary alcohol biosynthetic process,” and “steroid biosynthetic process” categories (Figure 4A). Moreover, KEGG pathway analysis showed that these DEGs were mainly enriched in “steroid biosynthesis” and “terpenoid backbone biosynthesis” pathways (Figure 4B). These results suggested that the HFD treatment primarily affected lipid synthesis and metabolism. Our results are consistent with previous studies which also revealed lipid biosynthetic and lipid metabolic process were enriched in the biological process (Chen et al., 2021; Xu et al., 2021). These results indicate a large proportion of dysregulated protein-coding genes in the transcriptome of a NAFLD liver, associated with glycolipid metabolism. However, there is no study exploring the effect of EMP on HFD-induced NAFLD.

Thus, we established HFD-EMP group to further analysis the potential biological processes. We identified 123 DEGs that were significantly changed in the EMP group, compared with the HFD group. GO enrichment analysis showed that the DEGs were mainly involved in “response to redox state,” “isoprenoid biosynthetic process,” “isoprenoid metabolic process,” “terpenoid biosynthetic process,” “steroid metabolic process,” and “sterol biosynthetic process” categories (Figure 4C). The KEGG pathway analysis indicated that these DEGs were mainly enriched in “terpenoid backbone biosynthesis” and “JAK-STAT signaling” pathways (Figure 4D). These results indicated that EMP mainly affects lipid oxidation and metabolism.

### PPI Network and GSEA Analysis

**FIGURE 5:**
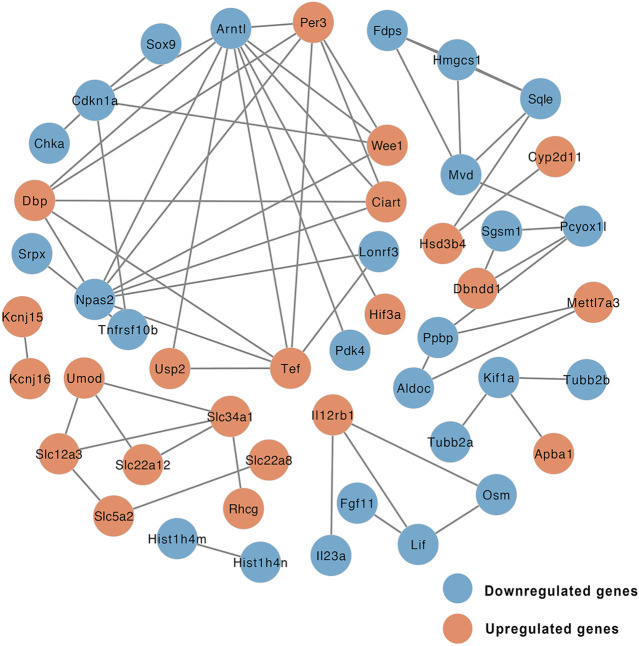
Network of protein-protein interaction (PPI) of DEGs between EMP and HFD group (orange node: upregulated genes, blue node: downregulated genes).

Gene expression data and PPI networks were used in combination to identify the regulated parts of the network in the drug treatment. This process created a PPI network involving DEGs of the EMP and HFD groups, such that the edges connecting co-expressed genes were preserved. To identify the “communities” and “hubs” of the subnetworks, we analyzed the differentially expressed parts of the transcriptome. This analysis revealed multiple communities that contained either up- or downregulated genes associated with EMP treatment (Figure 5 **)**.

**FIGURE 6:**
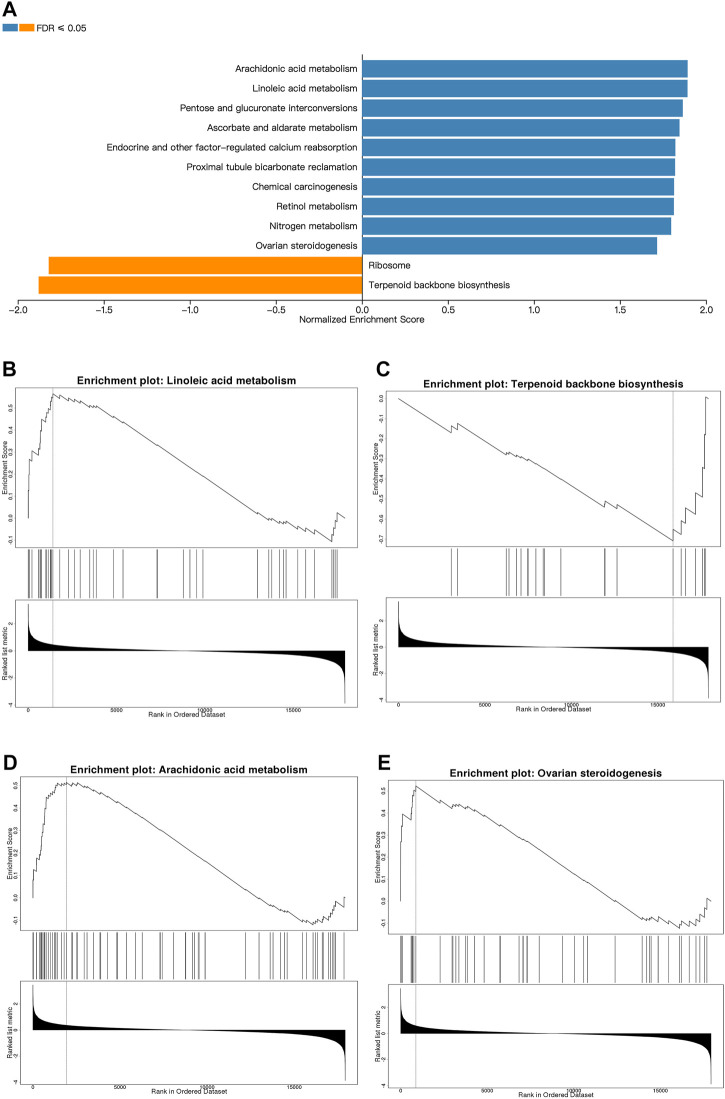
Metabolic pathways affected by EMP. **(A)** The top Reactome gene sets that are up- and down-regulated with respect to EMP treatment based on normalized enrichment scores (NES) for EMP/HFD genes. **(B–E)** Pathways related to lipid metabolism and synthesis which are selected from **(A)**. (orange: upregulated pathways, blue: downregulated pathways, FDR ≤0.01).

Next, we used GSEA to identify the differentially expressed pathways associated with EMP treatment. Although PPI network analysis identified co-regulated gene communities, GSEA showed additional pathways that were differentially associated with histological severity. In total, 12 pathways were affected by EMP treatment **(**FDR <0.05, Figure 6A). The full set of pathways identified in this experiment is provided in Table 3. Four pathways were associated with lipid metabolism and synthesis: “ovarian steroidogenesis” (upregulated), “arachidonic acid metabolism” (upregulated), “terpenoid backbone biosynthesis” (downregulated), and “linoleic acid metabolism” (upregulated) (Figures 6B–E).

**TABLE 3:** The full set of pathways identified.

Gene set	Description	ES	NES	*p* value	FDR
mmu04913	Ovarian steroidogenesis	0.52	1.72	0.00	0.04
mmu00590	Arachidonic acid metabolism	0.51	1.89	0.00	0.04
mmu03010	Ribosome	-0.49	-1.82	0.00	0.03
mmu00900	Terpenoid backbone biosynthesis	-0.71	-1.88	0.00	0.03
mmu04961	Endocrine and other factor-regulated calcium reabsorption	0.57	1.82	0.00	0.03
mmu00591	Linoleic acid metabolism	0.56	1.89	0.00	0.02
mmu00053	Ascorbate and aldarate metabolism	0.63	1.85	0.00	0.02
mmu04964	Proximal tubule bicarbonate reclamation	0.67	1.82	0.00	0.02
mmu00040	Pentose and glucuronate interconversions	0.62	1.87	0.00	0.02
mmu00910	Nitrogen metabolism	0.70	1.80	0.00	0.02
mmu05204	Chemical carcinogenesis	0.48	1.81	0.00	0.02
mmu00830	Retinol metabolism	0.48	1.81	0.00	0.02

### EMP Reduced Lipid Metabolism Through Increasing TG Lipolysis and Mitochondrial β-oxidation

**FIGURE 7:**
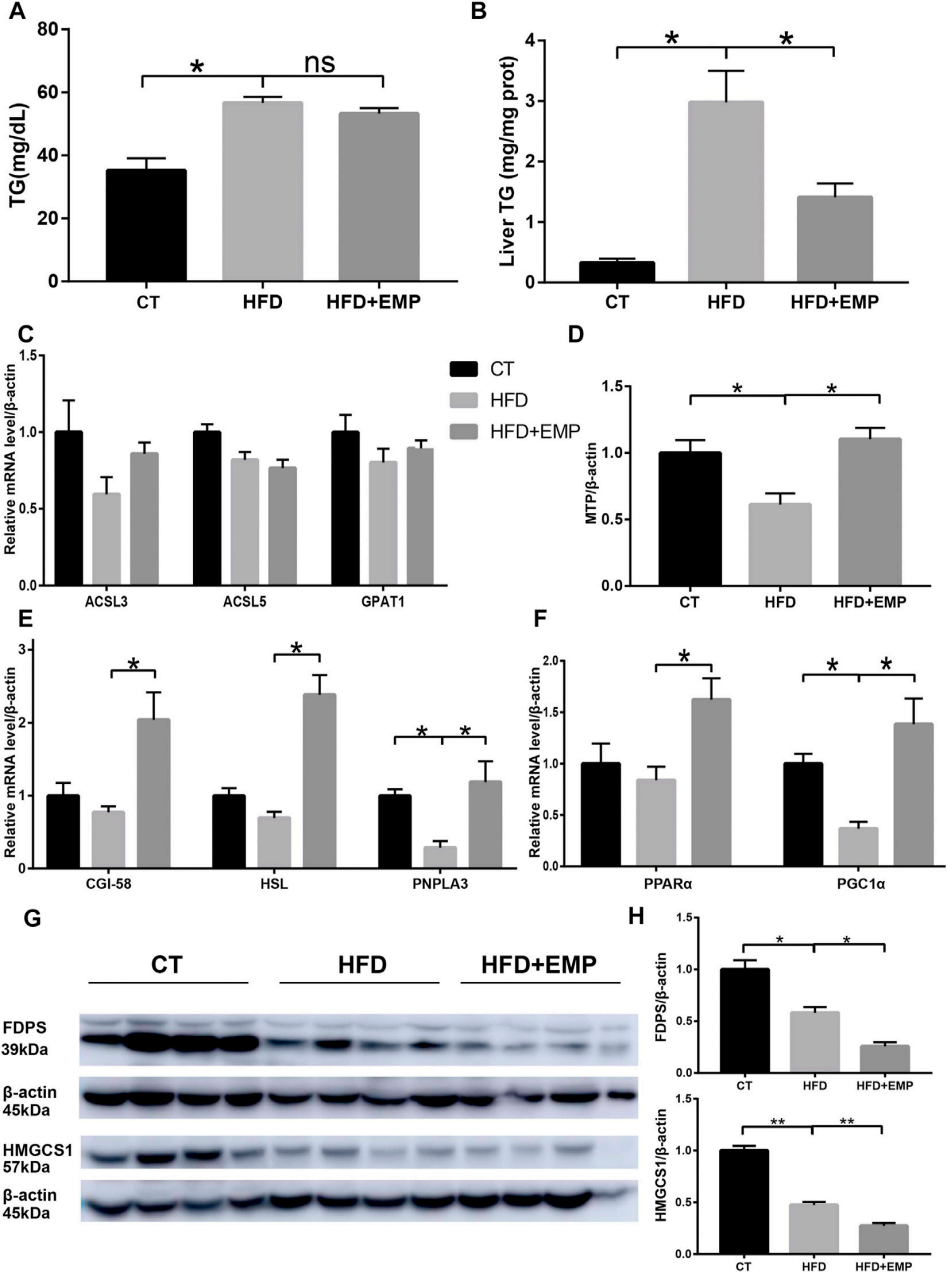
Empagliflozin reduced triglyceride level both in serum **(A)** and liver **(B)** through enhanced triglyceride transfer **(D)**, lipolysis **(E)** and microsomal mitochondrial β-oxidation **(F)**. Empagliflozin had no effect on the synthesis genes of triglyceride **(C)**. Western-blot analysis showed the protein levels of FDPS and HMGCS1 **(G and H)**, which were the most relevant genes in lipid metabolism. Equal loading of protein was verified by probing β-actin. Data represent means ± SEM. *p < 0.05, **p < 0.01, n = (4–6).

Consistent with previous studies, HFD mice showed significantly high TG content both in serum and liver (Figure 7A/B, Table 2), however, there was a decrease tend between HFD mice and EMP mice but no significant difference (*p* > 0.05). To further explore the underlying triglyceride molecules among groups, we detect the genes expressions of triglyceride metabolism and transport. As showed in Figure Figure7C, EMP had no effect on these genes (ACSL3, ACSL5, GPAT1) involved in the synthesis of TG. Compared with control group, HFD mice had lower expression of MTP, CGI-58, HSL, PNPLA3, meaning decreased triglyceride transfer and TG lipolysis (Figures 7D,E). However, these effects were reversed by EMP treatment. Moreover, EMP also enhanced these decreased-mitochondrial β-oxidation factors (including PPARα, PGC1α), which were overtly suppressed by HFD (Figure7F). To further explore whether RNA-seq data was consistent with protein level, we choose the most relevant genes in lipid metabolism to detect protein level through Western Blot analysis. As expected, the protein level of FDPS and HMGCS1was downregulated in mice after treatment of EMP (Figures 7G,H).

### qPCR Assessment of DEGs

**FIGURE 8:**
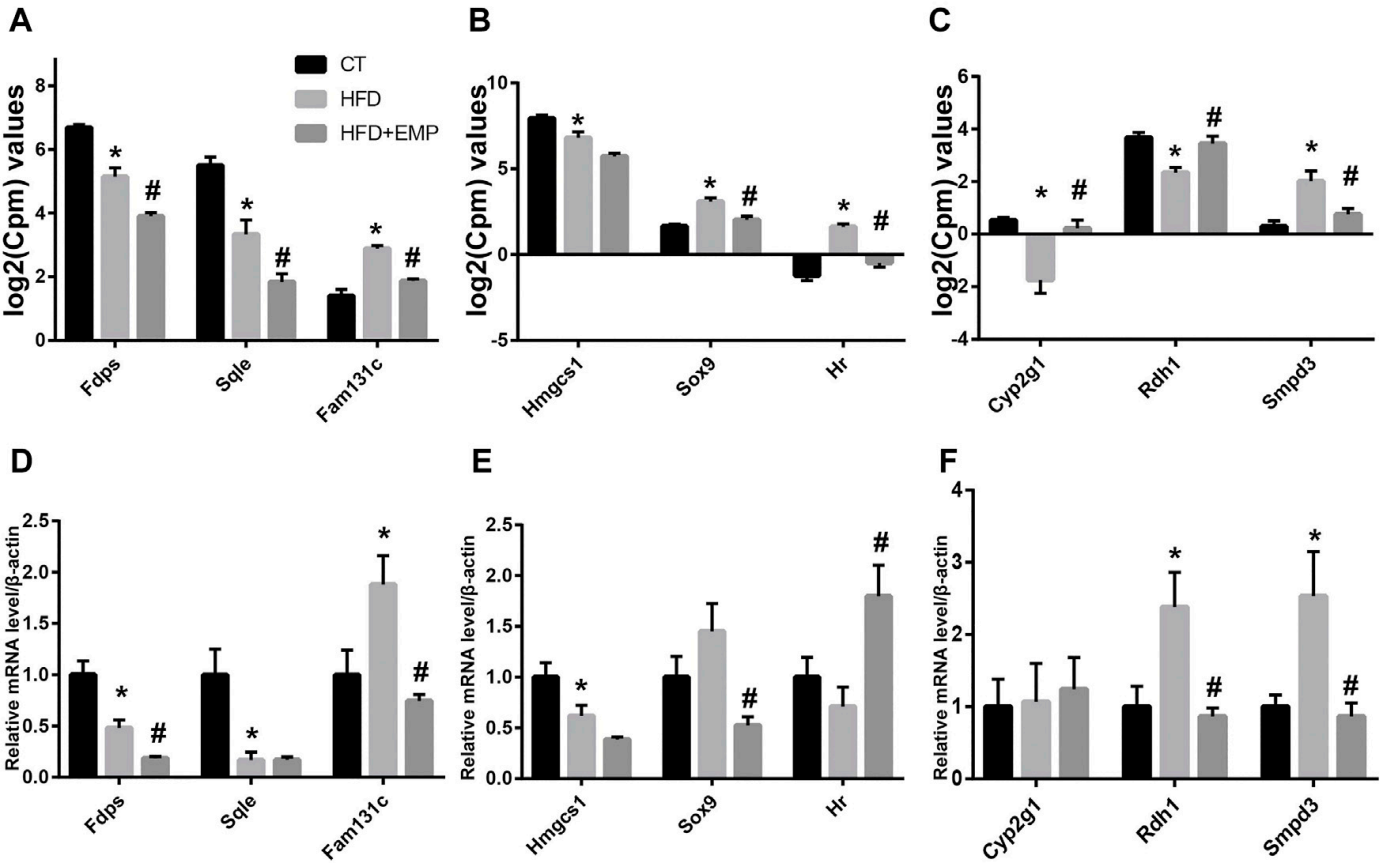
Quantitative real-time PCR validation of RNA-seq analysis of DEGs. Graphs represent log_2_(CPM) values of gene expression levels **(A-C)** in RNA-seq data and mRNA levels normalized to β-actin, *p < 0.05 vs. CT, #*p* < 0.05 vs. HFD, *n* = 5, **(D-F)** from quantitative real-time PCR data. Data are presented as means ± SEM, *p < 0.05 vs. CT, #*p* < 0.05 vs. HFD, *n* = (4–6).

To confirm the gene expression levels observed in RNA-Seq analysis, we chose nine genes that were mainly associated with lipid metabolism and synthesis (*Smpd3, Fdps, Hmgcs1, Rdh1, Sox9, HR, Sqle, Fam131c, Cyp2g1*). qPCR results were generally consistent with the RNA-Seq findings (Figure 8A). As showed in Figure 8B, the mRNA expression of Fam131c, Sox9, Smpd3 was upregulated in HFD groups compared with control group, these effects were reversed after treatment of EMP. Moreover, we found the expression of Fdps, Hmgcs1, HR, Sqle were downregulated in HFD-induced mice, however, these results were not reversed in EMP group.

## Discussion

This study investigated the effects of EMP on NAFLD. We found that EMP attenuated liver fat content and improved lipid metabolism by regulating genes related to lipid generation, transfer, lipolysis and oxidation. Our results provide a snapshot of the transcriptionally regulated pathways affected by EMP treatment for NAFLD; they may be important candidate pathways for alleviating disease. Additionally, the results provide insights concerning how these pathways interact to inhibit disease.

EMP is a novel type of antihyperglycemic drug that inhibits glucose reabsorption in proximal renal tubules. In addition to its effects on glycemic status in patients with type 2 diabetes, it contributes to weight loss and body fat reduction (Sun et al., 2020; Szekeres et al., 2021). Recently, clinical trials have reported SGLT-2i had beneficial effects on NAFLD, as evidenced by the remarkably reduced ALT, AST, triglycerides, hepatic insulin sensitivity indices (Ranjbar et al., 2019; Kahl et al., 2020). Of note, in EMPA-REG OUTCOME trial and E-LIFT trial also showed that treatment with empagliflozin significantly reduced liver enzymes and liver fat (Sattar et al., 2018; Kim and Lee, 2020). It also increases energy consumption, thermogenesis, and expression of uncoupling protein one in brown fat (Xu et al., 2017). Importantly, EMP attenuates NAFLD by activating autophagy and reducing ER stress and apoptosis (Nasiri-Ansari et al., 2021). Thus, these effects have emerged as important underlying mechanisms in NAFLD development and progression. In the present study, we found that 8-weeks treatment with EMP reduced body weight and fat mass, while alleviating HFD-induced liver injury. These results confirm the protective effects of EMP in a model of NAFLD.

The liver is essential for lipid synthesis and regulation of lipid metabolism. Healthy liver function involves balanced adjustment of lipid metabolism through multiple biological processes. In NAFLD, the compensatory enhancement of fatty acid oxidation is insufficient to normalize the lipid level; it may promote cell and tissue damage, as well as disease progression, by enhancing oxidative stress (Ipsen et al., 2018). In our study, we found that EMP reduced triglyceride level both in serum and liver through enhanced triglyceride transfer, TG lipolysis and microsomal mitochondrial β-oxidation. Previous studies identified DEGs that were associated with NAFLD onset and progression. Moreover, some studies examined gene expression using transcriptome data from liver biopsies of affected patients (Wruck et al., 2015). The findings revealed that many functional pathways and genes exhibited significant associations with NAFLD; many of these pathways and genes were related to lipid metabolism. Here, we used RNA-Seq to investigate the major pathways and genes involved in the effects of EMP on lipid synthesis and metabolism. Subsequent bioinformatics assays identified 123 DEGs with significant changes in expression levels after EMP treatment.

A previous study confirmed that overexpression of sex-determining region Y-box 9 (SOX9) enhanced lipogenesis and expression of PPARγ in sebocytes (Shi et al., 2017). In addition, HR lysine demethylase and nuclear receptor corepressor (HR) may be a adipogenic transcription factor by regulating the expression of PPARγ to generate white adipogenesis (Kumpf et al., 2012). PPARγ is the primary inducer of adipogenesis (Li et al., 2018) and regulates adipocyte differentiation. In our study, SOX9 mRNA levels were significantly reduced in the EMP group. We speculate EMP treatment inhibited SOX9 expression, which led to reduced lipogenesis in the liver. These changes reduced PPARγ expression, thereby inhibiting adipocyte differentiation. Sphingomyelins (SMs) have been reported as potential biomarkers for diagnosis of hepatic steatosis (Li et al., 2014). Additionally, a transcriptome analysis involved in mouse models of NAFLD and liver tissues from patients had reported the expression of SOX9 and SMPD3 (Teufel et al., 2016). In this study, sphingomyelin phosphodiesterase 3 (SMPD3) was inhibited in the EMP group, which may have led to increased SM expression. This finding conflicts with the results of a previous study (Zhang et al., 2019), in which SMPD3 had a lower expression level in tyloxapol-treated mice, then increased after treatment. This inconsistency is presumably because the animal models were established using different methods. The final biosynthetic product of the 3-hydroxy-3-methylglutaryl-CoA synthase 1 (HMGCS1) pathway is cholesterol. HMGCS1 is upregulated by galectin-7 (Gal-7); subsequent HMGCS1 activity increases the accumulation of cellular cholesterol (Fujimoto et al., 2021). Squalene monooxygenase (SQLE) is a critical regulatory point in the cholesterol synthesis pathway (Howe et al., 2017). Farnesyl diphosphate synthase (FDPS) and cytochrome P450 family 2g1 (CYP2G1) are both associated with lipid and cholesterol biosynthesis (Wang et al., 2017). In the present study, the expression levels of HMGCS1, SQLE, and FDPS were highest in the CT group and lowest in the EMP group. These gene expression differences provide important insights into the mechanisms by which EMP affects the liver. Family with sequence similarity 131c (FAM131C) is a group of human N-myristoylated proteins; protein N-myristoylation is required for proper targeting of SAMM50 to mitochondria (Takamitsu et al., 2015). Some studies have shown that overexpression of SAMM50 enhances fatty acid oxidation and reduces intracellular lipid accumulation (Li et al., 2021). In contrast, we found that the *Fam131c* expression level increased in the HFD group but decreased in the EMP group. Retinol dehydrogenase 1 (RDH1) is one of several enzymes that catalyze the conversion of retinol into all-trans-retinoic acid (atRA). RDH1 can suppress adiposity by promoting brown adipose generation (Krois et al., 2019). In this study, RDH1 expression levels differed between RNA-Seq and qPCR, presumably because of differences in the technical protocols.

Overall, we found that EMP treatment could reverse the activities of NAFLD-associated pathways. These transformations mainly involved reduction of lipid metabolism and synthesis pathways in the liver. Our results indicate that EMP treatment alleviates fat accumulation and slightly affects inflammation in NAFLD. GO and KEGG enrichment were performed to analyze these DEGs, with the aim of clarifying the mechanism by which EMP affects lipid synthesis and metabolism in the liver. Our findings showed that EMP mainly affects the response to redox state, triglyceride transfer, TG lipolysis and microsomal mitochondrial β-oxidation, and JAK-STAT signaling pathways. Importantly, EMP reduced lipid accumulation and alleviated pathological changes involved in NAFLD. These changes may be related to the regulation of lipid oxidation-associated gene expression by EMP. However, we only established the relation between mechanisms of EMP and NAFLD, but which cell type (hepatocytes, macrophages or hepatic stellate cells) in liver for lipid metabolism and synthesis is not determined in this work. Therefore, we will focus this limitation in the next research.

In summary, our results provide snapshot of the transcriptionally regulated pathways affected by EMP treatment for NAFLD; these may be important candidate pathways for treatment to slow the progression of NAFLD. Additionally, our findings provide insights concerning how these pathways interact to inhibit disease. Our research provides new insights concerning the mechanisms by which SGLT inhibitors impact NAFLD, particularly in terms of liver metabolism. The genes identified in this experiment provide robust evidence for further analyses of the mechanism by which EMP impacts NAFLD.

## Data Availability

The datasets presented in this study can be found in online repositories. The names of the repository/repositories and accession number(s) can be found below: https://www.ncbi.nlm.nih.gov/, PRJNA770493.
