# Supplementary figures and images for: Transcriptomic Analysis Reveals the Protective Effects of Empagliflozin on Lipid Metabolism in Nonalcoholic Fatty Liver Disease
Source: Front Pharmacol. 2021 Dec 21;12:793586. doi: 10.3389/fphar.2021.793586 (PMC8724565; doi:10.3389/fphar.2021.793586)

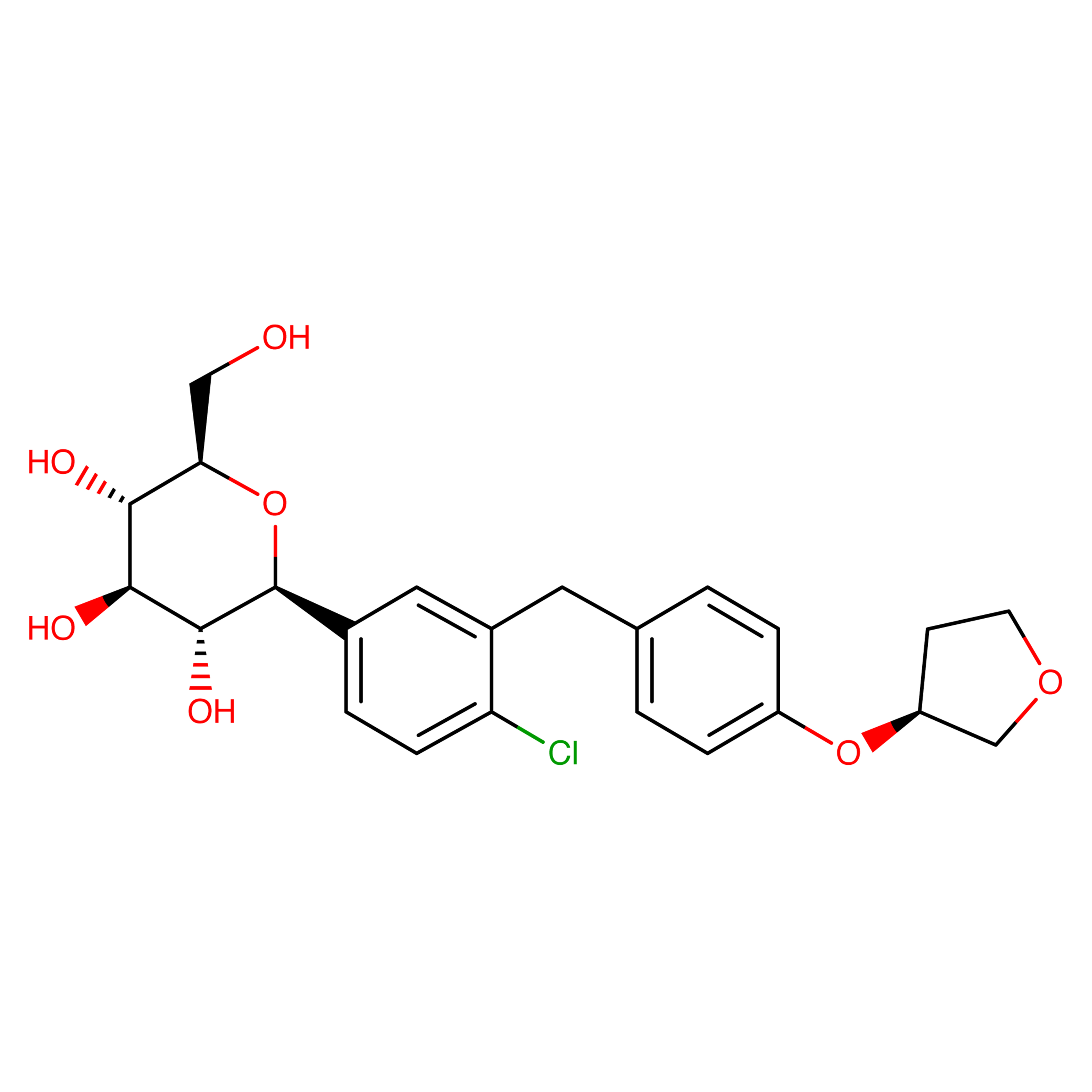

Supplement: Supplementary file 1 [file Image1.JPEG]
